# Case Report: Clinicopathological findings of a cutaneous mast cell tumor in a young domestic meerkat (*Suricata suricatta*)

**DOI:** 10.3389/fvets.2025.1706589

**Published:** 2025-12-03

**Authors:** Marco Graziano Castaman, Margherita Orlandi, Martina Baldin, Maria Massaro, Mattia Ridolfi, Maristella Zambelli

**Affiliations:** 1 Private Veterinary Laboratory—MyLav, Milan, Italy; 2 Department of Comparative Biomedicine and Food Science, University of Padua, Padua, Italy; 3 Clinica Veterinaria Casalpalocco SRL, Rome, Italy

**Keywords:** cytology, exotic pet, histochemical staining, histology, skin

## Abstract

A 10-month-old female domestic meerkat (*Suricata suricatta*) was presented with a cutaneous nodule in the left axillary region. The animal was clinically healthy and radiographs showed no evidence of involvement of visceral organs. Based on fine-needle aspirate that revealed numerous well-differentiated mast cells, a mast cell tumor was suspected. Surgical excision was performed and the mass submitted for histologic examination. Histopathological findings showed a round cell tumor, with marked cellular atypia and moderate mitotic activity; Giemsa special staining revealed a large number of fine cytoplasmic metachromatic granules. Histological and histochemical findings confirmed the cytological suspicion of a mast cell tumor. Based on prognostic factors used in the canine species, immunohistochemistry with antibodies against Ki67 (a marker of cellular proliferation) and CD117 (a KIT receptor localization marker) revealed that 90% of the neoplastic population was positive for CD117 (with a membranous staining pattern), and the Ki67 index was 28. The patient did not show local recurrence and sign of distant metastases at the 10 months follow-up. Based on literature search no previous reports of mast cell tumour in meerkats were retrieved. Therefore, this case highlights a previously undocumented presentation of mast cell tumor in this species, contributing to the expanding knowledge of neoplastic conditions in exotic companion animals.

## 1 Introduction

Cutaneous mast cell tumors (MCTs) are Cutaneous mast cell tumors (MCTs) are among the most common neoplasms in domestic animals, particularly in dogs and cats, with rare occurrences in horses, cattle and pigs (1). These tumors, though uncommon, have also been reported in exotic and non-traditional mammals, including ferrets and hedgehogs, as well as in reptiles and birds (2–4).

This neoplasm is well-documented and studied in its cutaneous form, particularly in dogs, in which different prognostic parameters have been established in the literature in the last decades (5). To predict the biological behavior of the neoplasm, clinical (size, localization, presence of ulceration and staging), histological (two grading systems based on mitotic count and cellular differentiation), immunohistochemical (proliferative index with ki67, KIT patterns, proliferating cell nuclear antigen and argyrophilic nucleolar organizing region) and molecular (c-KIT mutations) features are the most common used in the clinical practice (5–7).

Meerkats (*Suricata suricatta*) are endemic small carnivorous mammal of southern Africa, belonging to the order Carnivora.

In some European countries, it is not classified among prohibited or dangerous species according to the national guidelines and has gained popularity as a pet in recent years. However, literature on diseases affecting both wild and captive meerkats is extremely limited, with few reports of infectious and non-infectious diseases (8). Spontaneous neoplasms in captive animals are rarely described, with examples including: hepatic and intrathoracic liposarcoma (9, 10), ovarian luteoma (11), renal nephroblastoma (12), mandibular carcinoma (13), hepatic carcinomas (14, 15).

This report presents a case of a cutaneous mast cell tumor with a cytological, histological and immunohistochemical description.

## 2 Case presentation

**Figure 1:**
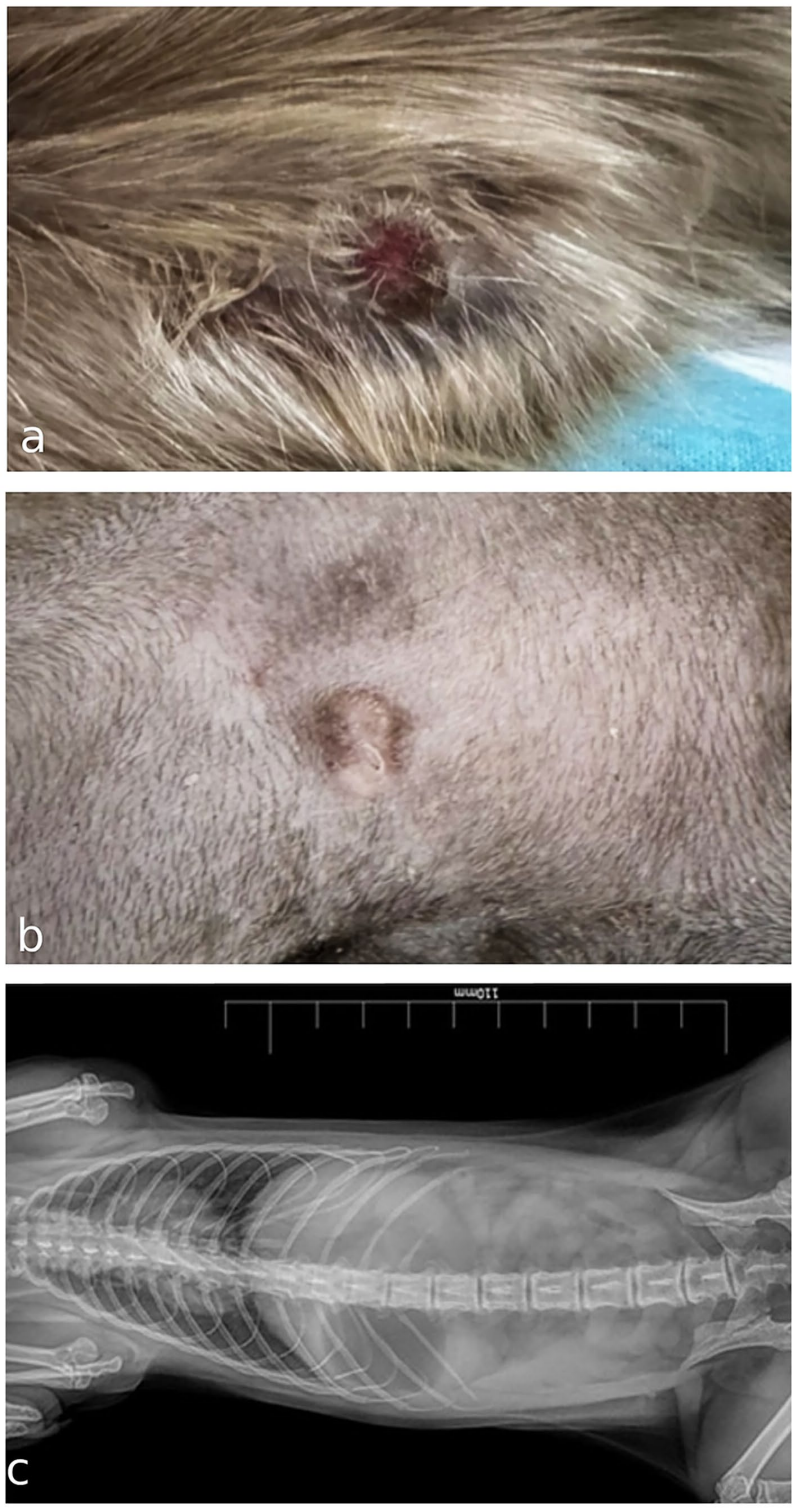
**(a)** Gross appearance of an ulcerated cutaneous mast cell tumor in the axillary region of a meerkat (*Suricata suricatta*) at first presentation. **(b)** Macroscopic appearance of the nodule after 2 weeks wearing a protective coat that reduces scratching and ulceration. **(c)** Ventrodorsal radiograph of thoracic and abdominal cavities in the absence of evident visceral lesions in a meerkat.

A 10-month-old intact female domestic meerkat (*Suricata suricatta*) was presented to a private veterinary practice with a cutaneous lesion in the left axillary region, first noticed one week earlier. Physical examination revealed a 0.5 cm diameter, elevated, poorly circumscribed, alopecic nodule with an ulcerated surface caused by constant scratching and biting (Figure 1a). After the application of a protective coat, scratching was reduced, and the lesion became less inflamed, but slowly growing (Figure 1b). The patient was otherwise clinically healthy, with no enlargement of regional palpable lymph nodes. Differential diagnoses included nodular skin lesions of inflammatory origin (bacteria, fungi, protozoa, or foreign body) or, less likely because of the young age, a neoplasm. Thoracic and abdominal radiographs showed no clear evidence of visceral lesions (Figure 1c). A fine-needle aspiration biopsy (FNAB) of the nodule was submitted to a private laboratory for cytology examination.

**Figure 2:**
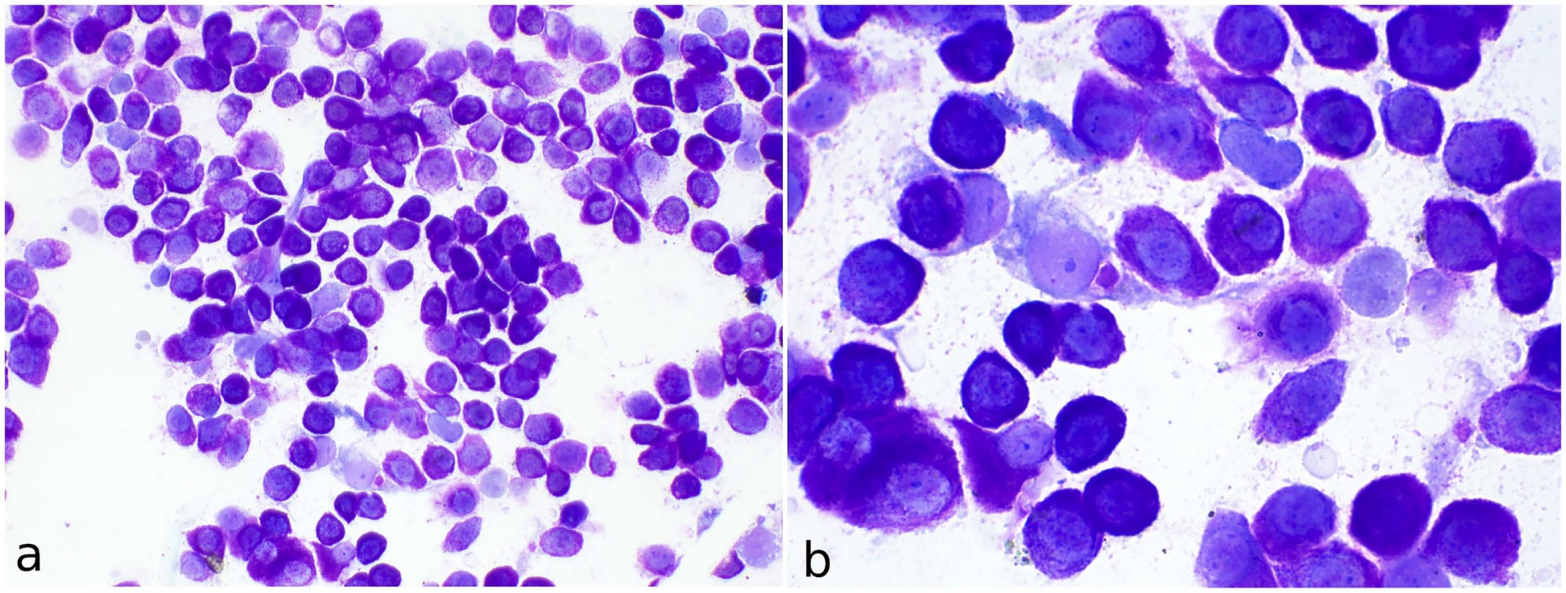
Fine-needle aspirate of a cutaneous mast cell tumor in the axillary region of a meerkat (*Suricata suricatta*). **(a)** May-Grünwald Giemsa quick stain (40x). The vast majority of cells consist of single well-differentiated mast cells. **(b)** MGG quick stain (100x) Numerous well-stained fine granules are visible in the cytoplasm of mast cells. Occasional binucleated mast cells and spindle cells are present.

A total of four cytological preparations were examined, following staining with a rapid May-Grünwald Giemsa method (MGG quick stain—Bioptica Milano S.p.A). The samples displayed moderate cellularity, with abundant blood contamination and numerous naked nuclei observed in the background. The predominant cell population consisted of round cells with uniform morphology, present either singly or in small to medium aggregates (Figure 2a). These cells exhibited moderate amounts of cytoplasm containing fine, metachromatic granules in moderate to high quantities. The nuclei were round to oval, eccentrically positioned, with granular chromatin and occasionally visible nucleoli (Figure 2b). Mild to moderate anisocytosis, mild anisokaryosis, and occasional binucleation were noted. Rare neutrophilic and eosinophilic granulocytes, along with occasional mononuclear cells containing phagocytosed basophilic cellular debris, identified as macrophages, were also observed. The round cells were identified as mast cells. The findings were deemed consistent with a diagnosis of mast cell tumor. A mixed nonspecific inflammatory process was considered as a differential diagnosis.

To perform an excisional surgery, the animal was premedicated with ketamine (5 mg/kg, Lobotor^©^, Acme Srl, Italy), dexmedetomidine (0.04 mg/kg, Dexdomitor^©^, Vétoquinol Italia Srl, Italy), and butorphanol (0.2 mg/kg, Torphadine^©^, Dechra Veterinary Products, UK), administered intramuscularly. An intravenous cannula was placed into the cephalic vein, and anesthesia was induced with propofol (2 mg/kg, PropoVet^©^, Zoetis Italia Srl, Italy) to allow orotracheal intubation. Anesthesia was maintained with isoflurane in pure oxygen, and continuous electrocardiographic monitoring was performed throughout the procedure. A wide area around the axillary region was clipped and aseptically prepared using a triple scrub technique alternating povidone–iodine solution and chlorhexidine.

A skin incision was made using a scalpel; electrocautery was avoided to prevent potential thermal artifacts in the tissue sample destined for histopathological evaluation. The mass was surgically excised with 1 cm lateral margins, while the deep margin was narrow due to the presence of muscular fascia. The incision was closed with a continuous intradermal suture using absorbable suture material. Postoperative management included antibiotic therapy with amoxicillin–clavulanic acid (12 mg/kg, PO, q24h for 7 days, Synulox^©^, Zoetis Italia Srl, Italy) and analgesia with meloxicam (0.2 mg/kg, PO, q24h for 3 days, Metacam^©^, Boehringer Ingelheim Animal Health S.p.A, Italy). Recovery from anesthesia was uneventful.

**Figure 3:**
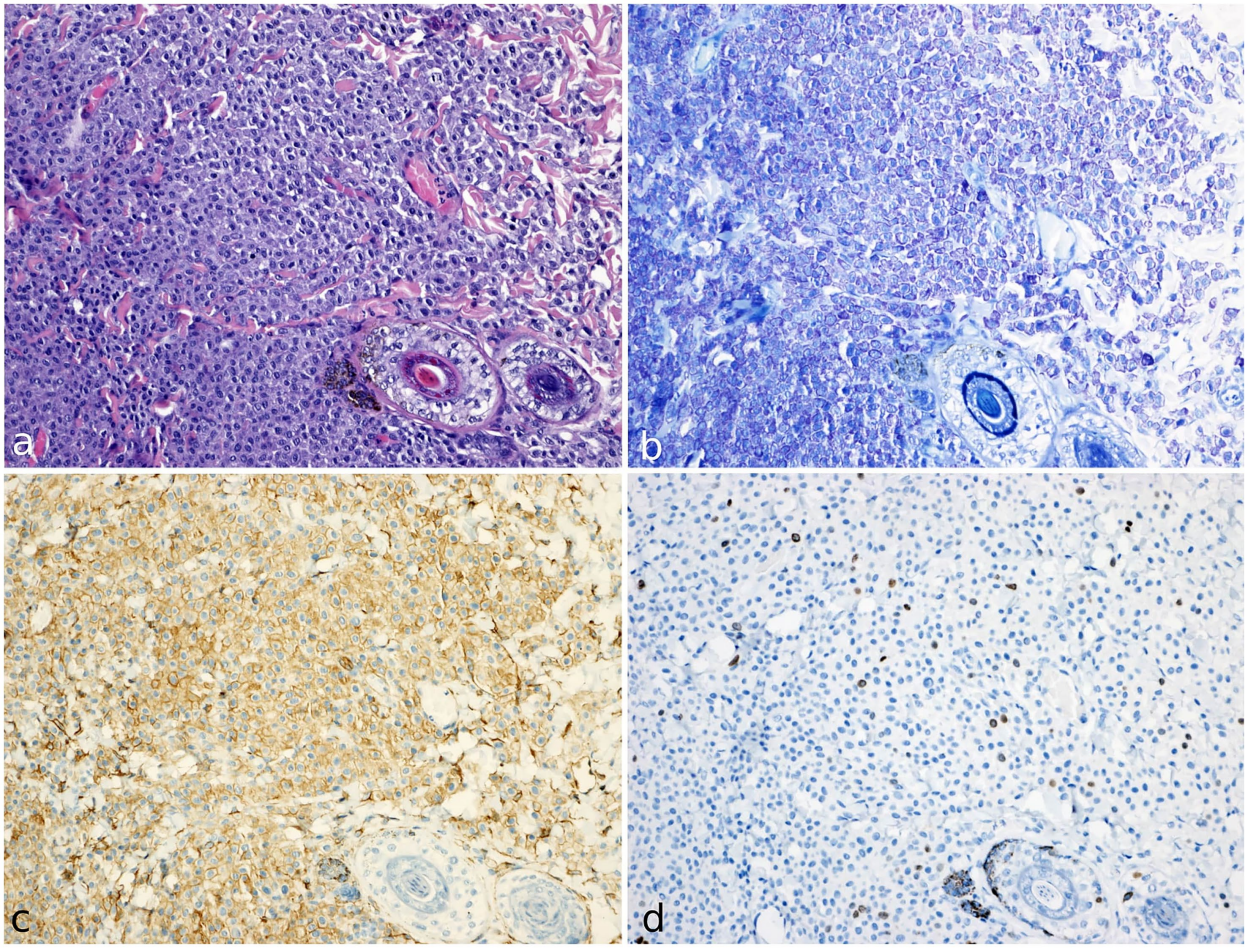
Histology, histochemical staining and immunohistochemistry of mast cell tumor in a meerkat (*Suricata suricatta*). **(a)** The cutaneous mass was composed of compact sheets of round cells. The cells show moderate amounts of amphophilic cytoplasm occasionally containing fine basophilic granules; anisocytosis and anisokaryosis were moderate to marked. H&E (20x). **(b)** Neoplastic population shows numerous cytoplasmic metachromatic granules. Giemsa staining (20x). **(c)** KIT receptor (CD117) immunostaining (20x). Cells express strong KIT immunolabeling localised on cytoplasmic membrane (pattern I). **(d)** ki67 immunohistochemical staining (20x). Numerous immunopositive cells are identified by brown nuclear staining.

The sample was then fixed in 10% formalin-buffered solution and submitted for histopathological evaluation. Histologic examination revealed a densely cellular, non-encapsulated, poorly demarcated neoplasm infiltrating the dermis and the subcutis (Figure 3a). The tumor consisted of sheets of neoplastic round cells 20–24 μm in diameter, dissecting preexisting collagenous stroma. The neoplastic cells had well-defined margins, moderate amounts of amphophilic cytoplasm occasionally containing fine basophilic granules, a round central nucleus with coarsely stippled chromatin, and 1–2 visible nucleoli. Anisocytosis and anisokaryosis were moderate to marked; the mitotic count was 9 per 10 consecutive high-power fields (HPFs; 2.37 mm^2^), with atypical mitotic figures. The overlying epidermis showed mild orthokeratosis. The excisional margins were assessed by cross-sectional technique, revealing focal infiltration of one lateral margin.

Giemsa staining confirmed the presence of numerous cytoplasmic purple metachromatic granules within the neoplastic cells (Figure 3b). The histological and histochemistry examination confirmed the cytological suspicion of mast cell tumor.

Given the lack of a specific grading system for mast cell tumors in meerkats, the two-tier grading system proposed by Kiupel and colleagues (9) for canine mast cell tumors has been adopted; considering the mitotic count > 7/10 HPFs, the neoplasm was accordingly classified as a high-grade mast cell tumor.

Based on prognostic factors used in canine species, immunohistochemistry with antibodies against Ki67 (Clone MIB-1; Dako Cytomation, United States) and CD117 (Kit protein; Dako Cytomation, USA) was performed to further characterize the growth fraction and KIT receptor localization of the neoplastic population, respectively (16).

Immunohistochemical analysis was conducted with an automated immunostainer Ventana Discovery Ultra (Ventana Medical System, Roche, United States), according to the manufacturer’s instructions. The antigen retrieval was performed with Discovery CC1 buffer (Roche, USA) and for antigen reveal the Discovery Chromomap DAB kit (Roche, United States) was used.

Cross-reactivity of the primary antibodies was assessed on non-neoplastic tissue adjacent to the tumor. Ki67 was evaluated within the basal layer of the epidermis and in the follicular bulbs, while CD117 showed positivity in dermal perivascular mast cells. As negative controls, sections from the same sample with omission of the primary antibodies were included (17).

Immunohistochemical analysis showed a strong immunopositivity for CD117, with membranous localization in over 90% of neoplastic cells (defined as pattern I) (Figure 3c) (18).

Ki67 immunolabeling showed nuclear positivity in the basal keratinocytes of the epidermis and the follicular bulbs and served as an internal control, demonstrating that these antibodies cross-react with this species. The Ki67 index was calculated as the average number of immunopositive cells in 5 random HPFs, in the areas of highest mitotic activity (16). The total number of immunopositive neoplastic cells was manually counted through the digital software ImageJ (National Institutes of Health, United States). The result of the Ki67 index was 28 (Figure 3d).

Despite the criteria of malignancy and the focal infiltration of the excisional margin, no further treatment was administered due to the lack of standardized protocols for treatment of mast cell tumors in meerkats. Surgical revision of the margins was not performed due to the anatomical site and further ancillary diagnostic investigations to exclude possible metastasis were not performed for financial reasons. Nevertheless, bi-monthly follow-ups were conducted, and at the last follow-up (10 months post-diagnosis), the patient exhibited no local recurrence or clinical evidence of distant metastasis.

## 3 Discussion

Mast cell tumor is a well-documented neoplasm in domestic species and reported in exotic animals, suggesting that mast cell proliferation disorders may occur across a wider range of taxa than previously recognized (2).

In ferrets, they are a common neoplasm, accounting for approximately one third of all reported cutaneous tumors. They occur more frequently in adult males and are generally benign, showing no local invasion or metastasis; surgical excision is generally curative (3, 19). Cutaneous mast cell tumors have also been reported in African hedgehogs, in which they can be classified as benign or aggressive according to the degree of cellular differentiation. Immunohistochemical analysis allows accurate diagnosis and can provide prognostic information, with well-differentiated forms being associated with longer survival following surgery (4).

The available literature on diseases affecting meerkats (*Suricata suricatta*) is extremely limited, and to date a comprehensive search of scientific databases has not evidenced existing descriptions of mastocytic neoplasia in meerkats (*Suricata suricatta*). A case of a cutaneous mast cell tumor in a young meerkat is described, highlighting its clinical, cytological, histopathological, and immunohistochemical features.

Unlike what has been reported in dogs and hedgehogs, where this type of neoplasia occurs more frequently in adult or elderly individuals, the meerkat in our case was young (10 months old) (4). A recent study has shown that in dogs less than one year old, both low- and high-grade mast cell tumors are associated with a better prognosis than in adults, suggesting that mast cell tumors may exhibit less aggressive behavior and that the standard prognostic criteria (Ki-67, mitotic index, grade) should be re-evaluated when applied to such young patients (20). Considering the absence of recurrence after almost one year follow-up, a similar clinical course can be hypothesized for the meerkat described in this case.

The cytological and histological findings are consistent with those reported in the canine species and differ from observations in ferrets, in which cytoplasmic granules are difficult to evaluate histologically (3). In the present case, although the cytoplasmic granules of the neoplastic cells were clearly evident in both examinations, Giemsa staining was performed to assess the metachromatic properties of mast cell granules thus confirming the mast cell origin of the tumor. This staining technique should therefore also be considered in meerkats as a useful additional diagnostic tool to differentiate poorly granulated mast cell tumors from other types of round cell neoplasms.

The diagnosis of a mast cell tumor in a species where this neoplasm has not been previously reported provides a unique opportunity for comparison with established cytological grading systems in dogs. In canine species, cytological grading systems are well-characterized and provide valuable prognostic information. Among these, the systems proposed by Camus et al. (21) and Scarpa et al. (22) are widely utilized. When applying the cytological criteria outlined by Camus et al., this mast cell tumor would be classified as well-differentiated and of low grade, with defining features including uniformity in mast cell morphology, the presence of abundant cytoplasmic granules, and minimal evidence of nuclear atypia or pleomorphism. Similarly, according to the cytological grading approach by Scarpa et al. (22), this tumor would also be categorized as low-grade. The criteria focus on factors such as the absence of mitotic figures, absence of multinucleated cells, and minimal anisocytosis and anisokaryosis, all of which align with the observed cytological characteristics in this case.

However, as this report describes a single case, any comparison across species should be interpreted with caution. Further cases are needed to determine whether these cytological criteria could be applied to meerkats or other exotic species.

These findings highlight similarities in cytological presentation between the mast cell tumor in meerkats and low-grade mast cell tumors in dogs. While direct extrapolation of prognostic implications may not be appropriate given the interspecies differences, the comparison underscores the utility of canine grading systems as a reference framework for cytological evaluation in other species.

On the other hand, histopathological examination highlighted moderate to marked anisocytosis and anisokaryosis and a mitotic count of 9 per 10 HPFs, indicating a high-grade tumor.

As in this case, some mast cell tumors described in dogs were characterized by disagreement between cytological and histological grading (21); therefore, we emphasize the importance of both diagnostic procedures for a more correct classification of a mastocytic neoplasia.

Moreover, in dogs, a ki67 index exceeding 10.6 or 23, as observed in this case, is correlated with higher mortality and greater risk of recurrence and development of metastasis, respectively (16, 23). In contrast, perimembranous KIT immunostaining (pattern I), as seen in the case here described, is associated with a more favorable prognosis in dogs, with low risk of recurrence, distant metastases and tumor-related death (18).

Compared to the canine species, the mitotic count >7 on 10 HPFs of this neoplasm and high ki67 index were both suggestive for a poor prognosis, whereas in contrast perimembranous KIT expression may predict a more favourable outcome; thus, a larger number of cases and additional studies are needed to assess the biological behavior of mast cell tumor in meerkats.

The animal was treated with surgical excision alone and, despite focal infiltration of the excision margins on histological examination, the animal showed no recurrence or evidence of metastasis at the 10-month follow-up.

## 4 Conclusion

This case represents the first documented occurrence of a spontaneous cutaneous mast cell tumor in a meerkat. Given its widespread occurrence in zoological institutions and the emerging popularity of this species as a pet, it is advisable to include this neoplasm in the list of differentials for cutaneous nodules. Further studies on a larger number of mast cell tumors in meerkats are required to properly elucidate the biological behavior of mast cell tumors and refine diagnostic and prognostic criteria for this species.

## Data Availability

The original contributions presented in the study are included in the article/supplementary material, further inquiries can be directed to the corresponding author.
